# A New Medical Dictionary

**Published:** 1847-12

**Authors:** 


ARTICLE XIX.

*A New Medical Dictionary;* containing an explanation of the terms in Anatomy Human and Comparative, Physiology, Practice of Medicine, Obstetrics, Surgery, Therapeutics, Materia Medica, Pharmacy, Chemistry, Botany, and Natural Philosophy, with the formulas of the principal pharmocopœias, and valuable practical articles on the treatment of disease. On the basis of Hooper and Grant. Adapted to the present state of Science, and for the use of Medical Students and the Profession. By D Pereira Gardner, M.D., Professor of Chemistry and Medical Jurisprudence in Philadelphia College of Medicine, etc. etc. 1 vol. 8vo. New York: Harper & Brothers. 1847. (From A, H. & C. Burley.)

This is a handsome volume about the size of Hooper's, got up in excellent style.

That our readers may understand the object of the publication, and the advantages it possesses over most works of its class, we allow the Editor to speak for himself, by quoting his preface entire.

"Dr. Hooper's Medical Dictionary has been, since its first appearance in London, a standard in the Profession. It has almost completely superseded the books of this class which were in circulation antecedently, and retains its original and imposing position. The publishers have not, however, overlooked the necessity of frequent emendations to keep pace with the rapid advancement of the medical sciences. The present seventh London edition has been completely revised and considerably improved by Professor Klein Grant, a gentleman of distinguished medical celebrity.

"Actuated by the same liberal motives, the American publishers have determined to keep pace with the improvements in the medical profession, and hence the republication of this work in a new and more compendious form. Adopting the last edition of the English work as a basis, the editor has bent his exertions, in this revision, to the production of a dictionary entirely adapted to the use of medical students, while he has endeavored to retain all the practical matter of the previous writers, so as to make it equally invaluable to the general practitioner. He has made an addition of many thousand articles, and more especially in the departments of chemistry, physiology, surgery, and the practice of medicine; nor has he lost any opportunity of giving notoriety to numerous American improvements, wherever the limits of the article have permitted."

